# COVID-19 in long-term care facilities in Frankfurt am Main, Germany: incidence, case reports, and lessons learned

**DOI:** 10.3205/dgkh000361

**Published:** 2020-11-05

**Authors:** Ursel Heudorf, Maria Müller, Cleo Schmehl, Stephanie Gasteyer, Katrin Steul

**Affiliations:** 1 Public Health Department of the City of Frankfurt am Main, Germany

**Keywords:** SARS-CoV-2, COVID-19, health department, nursing home, nursing home residents, long-term care facility LTCF

## Abstract

**Abstract:** As of August 30, 2020, the World Health Organisation (WHO) reported 24,822,800 COVID-19 infections world wide. Severe disease and deaths occur especially in older people with chronic illnesses. Residents of nursing homes are considered to be the most vulnerable group. In this paper, the experiences with COVID-19 in nursing homes in Frankfurt will be presented and discussed.

**Materials and methods:** Based on the data of the statutory reporting obligation, the reported COVID-19 cases are presented and incidences are calculated in different age groups and among residents of nursing homes. Outbreaks in various homes are described in detail based on the documentation from the public health department.

**Results:** By August 28, 2020, 2,665 COVID-19 infections were reported in Frankfurt am Main (incidence 351/100,000 inhabitants), including 116 (4.3%) residents of nursing homes (2,416/100,000 residents). Almost half (39%) of all deaths in Frankfurt (n=69; incidence 9.1/100,000) were among nursing home residents (n=27; incidence 558/100,000 nursing home residents), with 22 of them in just one long-term care facility (LTCF). Compared to previous years, the mortality rate in nursing homes did not increase in the first half of 2020. In one home, 75% of residents tested positive for SARS-CoV-2 and 25% died; in two other homes, 6.7% and 14.1% of the residents became infected, and the mortality rate was 0.5% and 1%, resp. In the other 42 homes in the city (3,906 beds), the infection rate remained below 1% and the death rate was 0.1%.

**Discussion:** In many countries, 30–70% of all deaths occur among nursing home residents, including Frankfurt (39%). An increase in overall mortality compared to previous years was not observed in Frankfurt as a whole or in the nursing homes in the city specifically. Due to the measures taken (monitoring of residents and staff, nursing care in protective clothing, prohibition or restriction of visits, physical distancing, isolation of infected people and quarantining of contact persons), only individual cases of COVID-19 illnesses occurred in nursing home residents in most homes and the outbreaks in the three homes could be stopped. We do not recommend regular nontargeted testing in nursing homes, but rather vigilance and the implementation of good hygiene as well as immediate targeted testing if COVID-19 is suspected in residents or staff. In order to mitigate the considerable negative effects of these measures on the residents, a good balance should be sought between infection prevention and the goal of ensuring self-determination and the residents’ quality of life.

## Zusammenfassung

**Abstract:** Bis zum 30.08.2020 meldete die Weltgesundheitsorganisation (WHO) weltweit 24.822.800 COVID-19 Infektionen. Schwere Krankheitsverläufe und Todesfälle treten besonders bei Älteren mit Vorerkrankungen auf. Bewohner von Altenpflegeheimen gelten als die vulnerabelste Gruppe. In der Arbeit sollen die Erfahrungen in den Altenpflegeheimen in Frankfurt diskutiert werden.

**Material und Methode**: Basierend auf den Daten der gesetzlichen Meldepflicht werden die gemeldeten Fälle dargestellt und Inzidenzen in verschiedenen Altersgruppen und bei Bewohnern von Altenpflegeheimen errechnet. Anhand der Dokumentation des Gesundheitsamtes werden Ausbrüche in verschiedenen Heimen detailliert beschrieben.

**Ergebnisse:** Bis zum 28.08.2020 wurden in Frankfurt am Main 2.665 COVID-19 Infektionen gemeldet (Inzidenz 351/100.000 Einwohner), darunter 116 (4,3%) bei Bewohnern von Altenpflegeheimen (Inzidenz 2.416/100.000 Altenpflegeheimbewohner). Nahezu die Hälfte (39%) aller Todesfälle in Frankfurt (n=69; Inzidenz 9,1/100.000) betraf Bewohner von Altenpflegeheimen (n=27; Inzidenz 558/100,000 Altenpflegeheimbewohner); davon 22 Bewohner in einem Heim. Im Vergleich mit den Vorjahren war die Sterblichkeit in den Pflegeheimen im ersten Halbjahr 2020 nicht erhöht. In einem Heim wurden 75% der Bewohner positiv getestet, 25% verstarben; in zwei weiteren Heimen traten bei 6,7% bzw. 14,1% der Bewohner Infektionen auf; die Sterblichkeit betrug 0,5% bzw. 1%. In den anderen 42 Heimen in der Stadt (3.906 Betten) blieb die Infektionsrate unter 1% und die Sterberate betrug 0,1%.

**Diskussion:** In vielen Ländern treten 30–70 % aller Todesfälle bei Altenpflegeheimbewohnern auf, auch in Frankfurt am Main (39%). Eine Erhöhung der Gesamtsterblichkeit im Vergleich zu den Vorjahren war in Frankfurt und in den Altenpflegeheimen in der Stadt nicht zu beobachten. Durch die ergriffenen Maßnahmen (Monitoring von Bewohnern und Personal, Pflege in Schutzkleidung, Verbot bzw. Einschränkung von Besuchen, *Social Distancing*, Isolierung von Infizierten und Quarantänisierung von Kontaktpersonen) traten in den meisten Heimen keine oder allenfalls Einzelfälle an COVID-19 Erkrankungen bei Altenpflegeheimbewohnern auf und die Ausbrüche in den drei Heimen konnten gut beendet werden. Statt ungezielter Testreihen empfehlen wir hohe Wachsamkeit, gute Hygiene und sofortiges gezieltes Testen beim Auftreten eines Verdachts auf COVID-19 als bessere Strategie. Um die erheblichen negativen Auswirkungen dieser Maßnahmen auf die Bewohner zu mildern, sollte im weiteren dringend eine ausgewogene Balance zwischen der Infektionsprävention und dem Ziel der Selbstbestimmung und Lebensqualität der Bewohner angestrebt werden.

## Introduction

As of August 30 2020, 24,822,800 confirmed cases of COVID-19, including 838,360 deaths were reported worldwide [1]. Although the majority of the infections are mild or even asymptomatic, – severe and fatal cases occur, especially in the vulnerable group of the older population with pre-existing chronic diseases. 

In Europe, the highest incidences of COVID-19-related deaths per 100,000 inhabitants have been reported from Spain (62.0), Italy (58.6), and Sweden (57.6). The lowest COVID-19 mortality has been reported from Iceland (2.9), Norway (4.8), and Finland (6.0), with Germany (11.0) and Denmark (10.7) ranging in between [[1], as of August 30, 2020].

Residents in long-term care facilities (LTCFs) are considered the most vulnerable population. About 30–66% of all deaths occur in this setting, as reported from several European countries (as of the end of May 2020) [2] and Canada, the USA and Switzerland [3], [4], [5]. High percentages of cororavirus-related deaths in LTCFs compared to the total population have been described in countries with very different incidences of COVID-19 infections and COVID-19 deaths, such as Spain (66%, 939.5 infections and 62.0 deaths per 100,000) or Norway (59%; 194.4 infections and 4.8 deaths per 100,000). In Germany, 38% of all COVID-related deaths occured in LTCFs [[2], as of May].

COVID-19 outbreaks in nursing homes have been reported from many countries. Up to three quarters of the residents of nursing homes who tested positive for SARS-CoV-2 were asymptomatic at the time of sampling, and up to a quarter of the residents died [6], [7], [8], [9], [10], [11], [12]. These asymptomatic transmissions were called the Achilles heel of the current strategies to control COVID-19 [13], which led to the demand for regular serial testing in nursing homes of both residents and staff [8], [9], [14], [15], [16], [17]. 

Complaints came from many countries. Nursing homes for the elderly felt they had been forgotten during the COVID-19 pandemic, reporting severe shortages in staff and personal protective equipment (PPE), receiving insufficient support and confronted with sometimes counterproductive government regulations [18], [19], [20], [21], [22], [23], [24]. 

There were many negative reports in the media about the situation in nursing homes [25]. The often problematic situation in nursing homes has been discussed as a result of long disregard and neglect of this sector [26]; the question “Could we have done better with COVID-19 in nursing homes?” was raised [27]. 

Various organizations published recommendations for the prevention of COVID-19 in nursing homes [28], [29], [30], [31], [32]. Essential elements are close monitoring of residents and staff with regard to suspected COVID-19 symptoms or anamnestic evidence of contacts with infected people through questioning, as well as some recommend observation of residents, including regularly taking their temperature. Small groups of residents should be organized and the same nursing staff continuously assigned to them, so that in case of an infection, as few residents as possible would require isolation and quarantine, and a minimum of employees would be quarantined in the event of an infection. Further organizational measures in everyday care should be obeyed: i.a., decentralized serving of the meals in the residents’ rooms or at staggered times in large, well-ventilated community rooms, suspension of community activities in the home, prohibition or at least restriction of visits by relatives and friends, use of personal protective equipment for care. Some guidelines further recommend the general wearing of mouth and nose protection. All guidelines demand immediate testing of residents or staff with signs of COVID-19 as well as their contact persons or the relevant area, and isolation of the respective resident and putting the employee on leave until COVID-19 is ruled out or until the end of the quarantine period.

In this paper, we report on the COVID-19 pandemic in Frankfurt am Main and the experiences from Frankfurt nursing homes with this situation. We present three outbreaks in three homes in Frankfurt as case studies and lessons learned. The data are discussed against the background of the increasingly controversial negative consequences of protective measures (isolation, physical distancing) on the health and quality of life of residents in nursing homes. Hence, a harmonious balance between infection prevention and independence and quality of life of the residents is necessary.

## Materials and methods

Frankfurt am Main, situated in the southwest of Germany, encompasses a population of 758,574 and hosts 45 LTCFs with a bed capacity for 4,835 residents. On March 2, the first inhabitants with COVID-19 disease were notified to the public health department after returning from holidays abroad. On March 6, the public health department informed all LTCFs of the imminent pandemic, and recommended physical distancing, omitting handshakes, obeying cough and sneeze etiquette, and frequent ventilation of the rooms. In a meeting on March 15, the directors of the LTCFs were informed about the current recommendations of the Robert Koch Institute (RKI) on COVID-19 in LTCFs [28], including hygiene measures, regulations for visitors, coping with shortage of personal protective equipment (PPE), quarantining of staff returning from holidays abroad, and handling of readmissions of residents from hospital stays. Recommendations were made to serve meals in the single rooms or at staggered times in large halls, regularly monitor the health of the residents and the staff, and build smaller staff units in order to be able to confine any introduction to smaller groups of residents and staff. In addition, since the middle of March, the Frankfurt public health department has provided protective material such as face masks when an absoulte shortage of these materials occurred.

In Hesse, the responsible ministry of social affairs ordered, among other things, a strict restriction of visitors of residents of LTCF on March 13, 2020. Visits by pastors, notaries and persons, who are to be granted access for professional reasons or because of sovereign tasks, were allowed, however. Only as of May 4 were visits allowed to a limited extent: one time per week for 1 hour, with monitoring of visitors for symptoms of COVID-19 and strict hygiene measures (physical distancing of 2 m, wearing a mask). As of June 22, visits 3x per week were allowed while maintaining the hygiene measures. This regulation is valid until October 15, 2020.

Based on data of mandatory reporting of SARS-CoV-2 and COVID-19 [33], we report the course of notifications in Frankfurt am Main in the general population as well as in residents and staff of LTCFs, including their symptoms. Incidence rates are calculated for different age groups in the population as well as for residents in LTCFs (based on the bed capacity available). In addition, the homes were asked to report the number of deaths per quarter from 2018 to 2020 on a voluntary basis. Case reports on outbreaks in three LTCFs are described and consequences (“lessons learned”) derived. 

## Results

### Notifications of COVID-19 cases, incidences, and symptoms in the general population and LTCFs 

**Figure 1:**
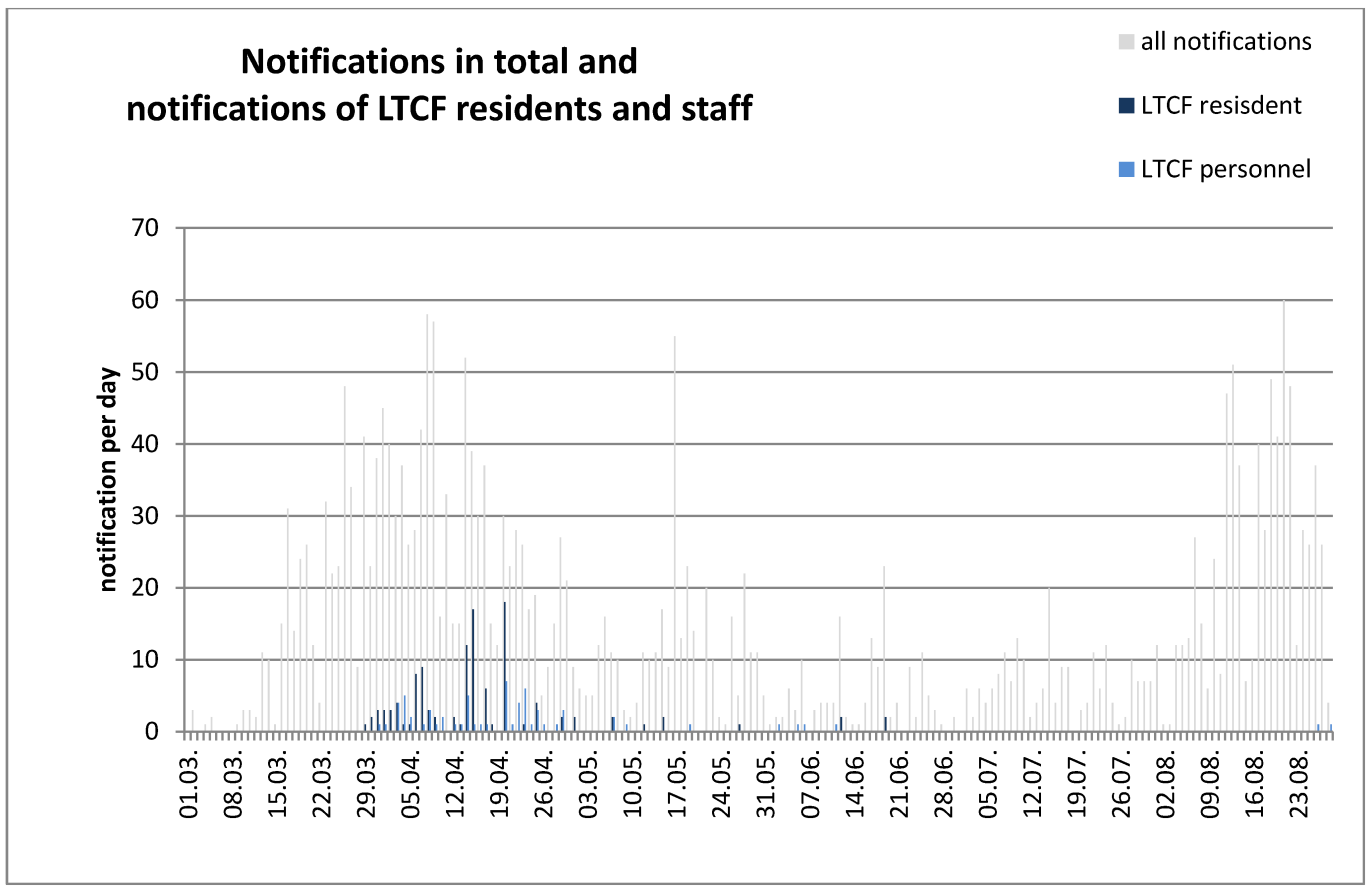
Notifications of persons infected with SARS-CoV-2 in Frankfurt/Main from March 2nd to August 28, 2020

**Figure 2:**
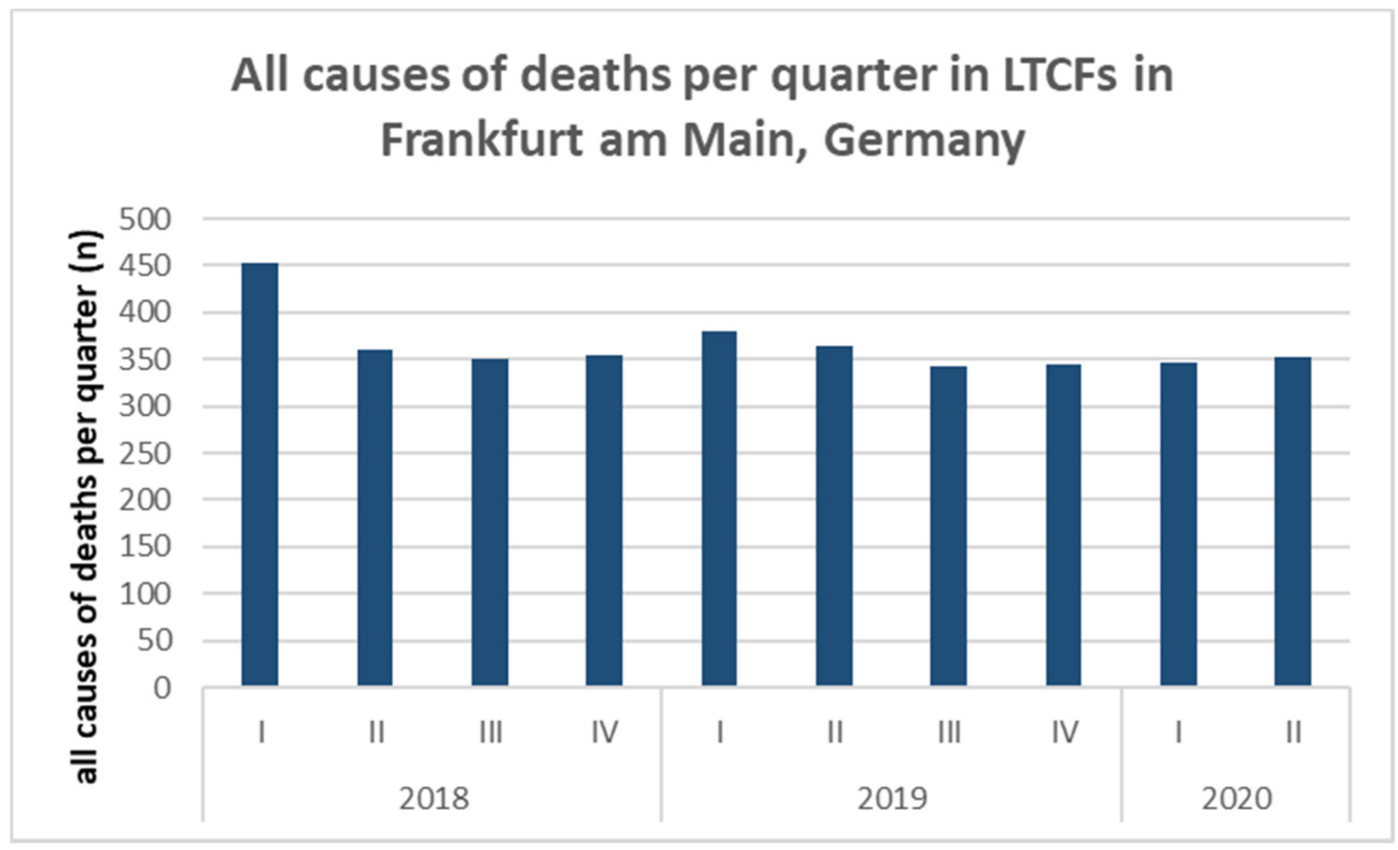
All causes of deaths in 40 long-term care facilities in Frankfurt am Main, quarterly data from 2018–2020

Figure 1 (Fig. 1) shows a high infection rate in Frankfurt from mid-March to the end of April. Afterwards, the reports decreased, increased again only in August, mainly due to travelers returning from foreign countries or large (family) gatherings. In April, many infections were reported among residents and employees of nursing homes. In the following weeks, only a few cases occurred in LTCFs and the last resident with COVID-19 was diagnosed on June 19. In July and August, no more infections were reported from LTCFs in Frankfurt, despite the sharp increase in total reports in August [34]. Total mortality per quarter as voluntarily reported by 40 LTCFs had not increased compared to previous years (Figure 2 (Fig. 2)). 

The population-related incidence for SARS-CoV-2 until August 30 was highest in young adults aged 20–39 years, followed by those aged 40–69 and those over 80 years old. Serious cases, which also resulted in hospitalization, and deaths showed a marked increase with age. The incidence of hospital admissions in people older than 80 years was 228/100,000, thus almost 4 times higher than in the general population (61/100,000); the incidence of deaths from or with SARS-CoV-2 was more than 10 times higher (in this age group) than in the total population (101 vs. 9/100,000) (Table 1 (Tab. 1)).

116 residents with SARS-CoV-2 were reported from long-term care facilities (LTCFs), which corresponds to an incidence of 2,416/100,000 care places based on the home care places in Frankfurt. 54 (47%) of these residents were admitted to hospitals (incidence of hospitalization 1,117/100,000 places) and 27 (23%) died (incidence of deaths from or with COVID-19: 558/100,000 places) (Table 1 (Tab. 1)).

Despite the high number of deaths from or with COVID-19 among LTCF residents, this did not exceed the mortality rates in the LTCFs compared to previous years (Figure 2 (Fig. 2)).

Compared with COVID-19 patients from the general population, LTCF residents with COVID-19 were more likely to be asymptomatic, less likely to have common cold symptoms, and no single resident reported loss of smell or taste. However, residents of LTCFs were more likely to suffer from severe respiratory symptoms. About half of the COVID-19 patients from nursing homes had to be hospitalized and about a quarter of them died. The hospitalization and death rate among nursing home residents was much higher than in the group of over-80-year-olds in the general population (including 58 nursing home residents; Table 2 (Tab. 2)).

### Case reports: COVID-19 in nursing homes

#### LTCF 1

Nursing home 1 has 89 residents. The general use of face masks for the personnel was implemented on March 23, 2020. However, there was still a general lack of protective equipment at this time. 

On March 27, 2020, a resident with symptoms suggesting COVID-19 was reported as a suspected case to the health department. Since the attending doctor was unable to test the resident in the home for SARS-CoV-2, the resident was immediately admitted to a hospital for diagnosis. When the positive result was reported on March 29, 2020, the health department carried out smear tests for SARS-CoV-2 on March 31 and April 1, 2020, on all residents (n=89) and employees (n=82). Due to the bottlenecks in the analytical facilities still existing at the time, it took almost a week before the results were available. A total of 10 residents (two in the affected living area, and 8 in the living area for dementia residents) and 6 employees tested positive. 

On Saturday, April 3, 2020, the neighbor in the bed next to the index case and on Sunday, April 5, 2020, two more residents fell ill and were admitted to a hospital.

On April 3, 2020, one of the employees who had cared for the index resident and who had been tested on March 31, 2020, was reported to be positive for SARS-CoV-2. Further investigation revealed that this employee had already developed symptoms on March 19, 2020, and visited her family doctor on March 20, 2020, who put her on sick leave without testing for SARS-CoV-2. The employee was back on duty from March 26 to March 29, 2020. The employer only became aware of the reason for the notification of illness on March 29, 2020. Then, the employee was immediately put on leave from work to prevent further spreading.

When on April 7, 2020, two more residents of another ward fell ill, it was decided that all previously negative residents of the whole institution should be retested for SARS-CoV-2. This was done by members of the German Red Cross (DRK) on seven days between April 14 and April 30, 2020. With more and more suspected cases arising, all residents were cared for as if they were COVID-19 patients from April 18, 2020, onward: i.e. single-room isolation and nursing care in protective equipment. The health department offered further information and help on April 21, 2020, and gave a practical training session on April 23, 2020, for employees on the early and late shifts on how to put on protective gowns, implement general and special hygiene, and especially perform hand disinfection, including practical exercises with fluorescent hand disinfection. As a consequence, the employee hygiene performance and behaviour improved significantly and the outbreak was stopped. A total of 67 (75%) residents and 29 employees were infected in this outbreak, and 22 residents died.

**Figure 3:**
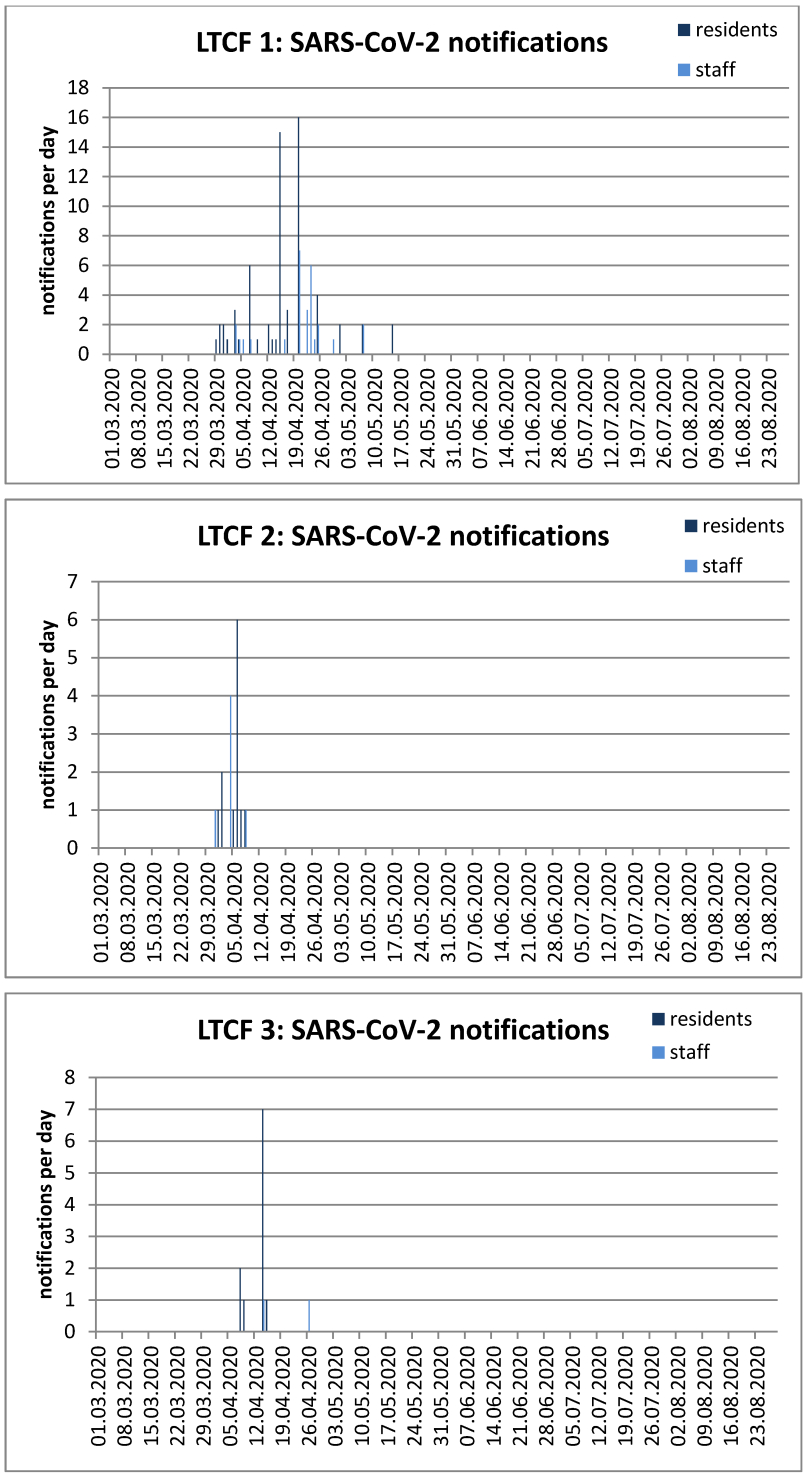
Figure 3 a–c: Sars-CoV-2-positive tested residents and staff in three long-term care facilities

Figure 3a (Fig. 3) shows the course of the COVID-19 outbreak in this facility. The data presented are the first time a positive test of residents and/or employees was reported. The outbreak started at the end of March and two residents tested positive on May 15. The infection route remains unclear. Most likely, the index person was a member of staff. Since then, neither residents nor employees have contracted COVID-19 (as of August 28). 

#### LTCF 2

This large LTCF offers accomodation and care for 209 residents, including 48 residents in “phase-F”, i.e. residents with severe neurological, mental, and emotional disorders despite intensive rehabilitation measures. The disabilities range from permanent unconsciousness and apallic syndrome to severe impairment of mental and/or physical functions, so that independent living is no longer possible. The home had switched to serving meals in the residents’ rooms and had implemented visitor restriction at an early stage. The obligation for personnel to wear a face mask was implemented on March 24, 2020. The health of the residents was monitored several times a day, and the health of the employees daily. The home was well equipped with protective equipment, including FFP-2 masks, for the care of the patients on the phase-F ward, but in the common units additional equipment was necessary. 

On March 29, 2020 the management of the home informed the public health department that an employee of an external employment agency and her co-worker, who had been working in the home until March 19, had visited an emergency-care physician for suspicion of COVID-19. Unfortunately, as the persons did not live in Frankfurt, neither the home nor the institution obtained any more information on their diagnosis. On March 31, 2020, the health department was informed that a night nurse on the phase-F ward had tested positive. Her last shift on this ward was on March 23, 2020, i.e. before the general obligation to wear masks. 

One day before that notification, on March 30, 2020, a resident became symptomatic for COVID-19 and was admitted to a clinic where he tested positive for COVID-19 on April 1, 2020. As he had visited residents on the phase-F ward, as of March 30 all residents of the unit affected first were cared for under isolation. 

On April 2, 2020, the health department tested all residents on the phase-F ward, the ward of the index patient (n=62) and the employees on site (n=70). A total of 6 employees and 14 (6.7%) residents tested positive. All of them lived or worked on the phase-F ward, and no person tested positive on any other ward. On April 8 and 9, 2020 all remaining residents and employees of the house (137 residents and 106 employees) were tested: all of them tested negative.

The outbreak could be limited to the phase-F ward and was ended very quickly, with the last case on April 5 (Figure 3b (Fig. 3)). The index case from the other ward died, but none of the bedridden and seriously ill residents of the phase-F ward did. The possible reason for the outbreak is assumed to be introduction of the virus by external employees without properly informing the director of the institution. 

#### LTCF 3

This facility hosts 99 residents, most of them in single rooms. The home was well equipped with personal protective equipment for the staff and did not report any need for additional material when the public health department started its survey near the end of March 2020.

On April 7, 2020, the home reported 3 residents with suspicion of COVID-19. Two of the symptomatic residents were immediately admitted to a hospital and tested negative for SARS-CoV-2. The third symptomatic resident remained in the facility; the test carried out immediately by the family doctor detected COVID-19 infection.

On April 12, 2020, 137 asymptomatic residents and 69 employees were tested for SARS-CoV-2 by the *Arbeiter Samariter Bund* (ASB). A total of 14 residents and 3 employees tested positive. Four residents were admitted to a clinic and one died.

The home management immediately implemented all necessary measures, including isolation and nursing care for all residents in their own room. Apparently, all residents complied well with this. Most of the residents readily cooperated; but residents with dementia could also be encouraged to do so. Thus, the outbreak ended quickly (Figure 3c (Tab. 3)). The route by which the virus was introduced to this institution could not be determined. 

Table 3 (Tab. 3) shows the comparison of these three LTCFs with the other LTCFs in Frankfurt. There are not only clear differences within the three homes in terms of infections, hospitalizations and deaths, but these three also differed from the others. Compared to 95 infections and 24 deaths of residents in these three LTCFs, in the remaining 42 homes in total, only 21 infections occurred among residents and 3 residents died.

## Discussion

As of August 30, 2,665 COVID-19 cases were reported in Frankfurt, including 463 hospitalizations and 69 deaths. The incidence corresponds to 351.3 infections/100,000 inhabitants and thus slightly exceeds the incidences in the federal state of Hesse (246/100,000) and Germany as a whole (291/100,000) [35]. In contrast, the deaths in Frankfurt amounted to 9.1/100,000 and were thus well below the national average of 11.2 deaths/100,000 inhabitants. In residents of nursing homes, however, the incidences of infections, hospitalizations and deaths (2,416, 1,116 and, 558 per 100,000 residents) were significantly higher than the incidences in the general population. This emphasizes the high risk of this vulnerable group in LTCFs.

Most infections, including hospitalizations and deaths, occurred in April 2020, after which there were only a few deaths. The overall mortality has not increased in any phase of the pandemic, neither in Frankfurt [34] nor in Hesse [36].

However, as described in many countries with different incidences of infections and deaths [1], [2], a large proportion of the deaths in Frankfurt also concerned residents of nursing homes: 27 of 69 (39%) deceased were residents of nursing homes. In one home (LTCF 1), 22 (24.7%) of the residents died. In many other homes in Frankfurt, however – under the extensive restrictions due to Corona – there were neither COVID-19 infections nor deaths. Taking all LTCFs together, 0.6% of residents died in connection with COVID-19. Nevertheless, the mortality rate did not exceed that of previous years, neither in the general population nor in the group of nursing home residents, despite the many deaths in LTCF 1. 

Communication and cooperation between the nursing homes in Frankfurt and the health department has been good for many years. The health department had developed a hygiene ranking for LTCFs from its on-site inspection [37]. Infection hygiene inspections by the public health department take place annually and as needed („bei Bedarf“). The inspections focus on various aspects, such as surface cleaning and disinfection, and handling of laundry or urinary catheters [38], [39], [40]. Many homes are very interested in hygiene issues, and some of them have participated in the HALT project (Healthcare-associated infections in long-term care facilities) dealing with infections and the use of antibiotics in nursing homes in Europe [41]. Many of them are members of the regional network on MDRO Rhein-Main [42], have taken part in studies on MDRO in nursing homes [43], [44], [45], and participate in the network’s various advanced training courses, encompassing hand hygiene, general hygiene measures and the safe handling of protective clothing [46]. Hence, the LTCFs were in a good position at the beginning of the pandemic. Infection control visits by members of the public health department during the pandemic (from May to August 2020) confirmed good conditions in hygiene and infection prevention in the nursing homes of Frankfurt [47].

As in many other studies [6], [7], [8], [9], [10], [11], [12], a large proportion (41%) of residents of nursing homes in Frankfurt am Main who tested positive were asymptomatic at the time of testing. Residents complained of general symptoms, cough, sore throat, etc. significantly less often than SARS-CoV-2 infected people in the general population. The typical symptoms such as loss of smell and taste were never reported by LTCF residents. Older people or people with dementia may be less able to express such symptoms. Nursing home residents, however, suffered more often from severe breathing difficulties and had to be admitted to a hospital and ventilated more frequently than COVID-19 patients in the general population. The fact that the employees of nursing homes who tested positive for SARS-CoV-2 reported COVID-19-specific symptoms more often and were less frequently asymptomatic than the COVID-19 patients in the general population can possibly be explained by the fact that they are well-informed specialists from the nursing field, competent in symptom observation. None of the employees with COVID-19 developed severe respiratory symptoms, only 2 (3%) were hospitalized and none died.

In its daily status reports, the Robert Koch Institute publishes numbers of infections, hospitalizations, and deaths of residents and employees in facilities according to the Infection Prevention Act § 23 (medical institutions), § 33 (schools and daycare for children), and § 36 (facilities for the care of older, disabled, or other persons in need of care, homeless shelters, community facilities for asylum seekers, repatriates and refugees as well as other mass accommodations and prisons) [35]. Since § 36 of the Infection Protection Act comprises not only inpatient care facilities but also other facilities for mass accommodation, the “supervised facilities according to § 36 of the Infection Protection Act” listed in the daily information of the RKI cannot be directly compared to the residents of nursing homes described here. In Frankfurt am Main, 127 residents in accommodations for asylum seekers (another large group of “people in institutions according to Section 36 of the Infection Protection Act”) tested positive; they were often asymptomatic or exhibited only a mild form of the disease, and none of them died [47].

The individual nursing homes in Frankfurt were affected differently by COVID-19 infections. At the beginning of the pandemic in Frankfurt am Main at the end of March 2020, there was still a lack of personal protective equipment (PPE) and testing capacity, and also some uncertainty in dealing with COVID-19 patients. The COVID-19 organizational and hygiene measures were not yet fully established. In a nursing home affected by COVID-19 at this early stage, 67 (75%) of the residents contracted COVID-19 and 25% died. 29 employees of that home also tested positive. The outbreak could only be stopped after a month. In two other LTCFs, 14 residents and 3 or 6 employees each tested positive; these outbreaks were stopped within a few days by the measures taken. No further SARS-CoV-2 cases have occurred in these homes as of the end of August 2020, neither among residents nor among employees. It cannot be ruled out that asymptomatic cases occurred and were not diagnosed. However, a greater spread with symptomatic infections or even hospitalizations can definitively be ruled out; the health department would have noticed this through the obligation to report positive tests. It remains unclear whether this encouraging situation is due to the effectiveness of the measures taken (visitor restrictions, physical distancing, etc.) or to the low overall number of infections in the region. In a US-American study, the strongest predictor of cases and outbreaks in nursing homes were the per capita cases in the country [48], [49].

Two points are also noteworthy: In 22 other LTCFs in Frankfurt am Main, isolated cases of COVID-19 were described among residents or employees, but the measures taken prevented wider spreading in these homes. The remaining 20 homes reported neither residents nor employees as infected with SARS-CoV-2, even though extensive tests were carried out in April 2020.

In numerous publications on COVID-19 in nursing homes in various countries and in recommendations from national authorities, regular testing in LTCFs (residents and staff) is recommended, with rapid turn-around times [6], [7]. In the LTCFs in Frankfurt am Main, at the beginning of the pandemic in the homes with individual COVID-19 cases, the residents of the affected wards and contact persons were tested for SARS-CoV-2 (targeted testing). In non-targeted test series in April 2020, 2,354 residents and 1,751 employees were examined in another 24 homes: A total of 5 residents and 3 employees (0.2% each) tested positive. In the following months, no more serial tests were carried out in nursing homes in Frankfurt. Up to the end of August, there were neither singular cases nor outbreaks in the LTCFs. Therefore, and because of the low test accuracy and frequent false positive results in situations with low prevalence of SARS-CoV-2 [50], [51], we conclude that regular testing in nursing homes is not necessary. Vigilance and the implementation of good hygiene as well as immediate testing if COVID-19 is suspected in residents or staff seem to be a better strategy. 

A closer look at LTCF 1 and 2 shows that it is essential for employees with symptoms to precisely and immediately inform the LTCF management. In both cases, SARS-CoV-2 was probably registered by employees, but the home management was informed too late about the employees’ health problems. In home 2, the infection process affected the high-risk phase-F ward with immobile residents in need of intensive care. In this situation, further dissemination is possible via employees only and can be easily prevented by good hygiene management. In LTCF 1, several wards were affected within a very short time period, including a living area of residents with dementia and a great need to move around and wander. Therefore, and because of uncertainties among the staff regarding the hygiene measures and the use of PPE, it was difficult to contain the outbreak. In home 3, which also houses many residents with dementia, the necessary isolation and hygiene measures were nevertheless effectively implemented and the outbreak ended quickly.

Overall, however, our experience and that of others [52] has shown that the recommended measures are effective: daily surveillance of both staff and residents and the ability to test quickly, universal PPE, physical distancing and limited traffic through LTCF by restricting visitors and other people. Also in agreement with Kim et al. [52] – despite recommendations by the CDC to the contrary – we consider targeted testing of residents or employees with COVID-19 symptoms sufficient and find routine series testing unnecessary. D’Adamo et al. [53] summarized the experiences and recommendations as ABCD: awareness (monitoring of residents and staff), behaviour (PPE, physical distancing), containment (proper hygiene and distancing for example in meetings and conferences), decisions (i.e. back up plans for potential staff shortages) [53].

Nevertheless, success in infection prevention comes at a high price. The negative consequences of the infection prevention measures for the residents are increasingly being discussed: immobility, deterioration in health, especially for people with dementia, social isolation, loneliness, depression, restriction of self-determination and quality of life and a restriction of end-of-life support with dignity [27], [54], [55], [56], [57], [58]. “The ‘Confinement Disease’ is propably more deleterious than the Coronavirus Disease 19 (COVID-19) itself” [59]. Recommendations for regular calls from relatives, making contact via video, simulated face-to-face therapy (video games and questions to the residents) [57] etc. can mitigate the negative consequences, but this is by no means sufficient. Thus it is encouraging that the relaxation of the ban on visits resulted in no infections in LTCFs, neither in the homes in Frankfurt am Main nor in 26 homes in Holland [60]. 

Employees in LTCFs are also affected by these negative consequences [27], [61]. Not only their physical but also their emotional stress has increased considerably. Help might be provided through better information and training. Other suggestions encompassed a specialist telephone hotline, out of hospital mobile geriatric teams, and regional COVID-19 videoconferences and support teams for multidisciplinary decision making [27]. But these also fall short, as the basic understanding of activating and promoting care for the elderly and of nursing homes as living places for residents is not taken into account.

At present, decisions for residents in nursing homes are made almost exclusively under the aspect of infection prevention. Looking ahead, the nursing home as a place of living for the residents and the residents’ self-determination and quality of life must again be placed at the forefront [58]. 

Therefore, the German Society for Nursing Science published the S1 guideline “Social participation and quality of life in inpatient care for the elderly under the conditions of the COVID-19 pandemic” on August 10, 2020 (DGP). Fourteen recommendations to improve the social participation and quality of life of the residents and 8 recommendations to support the employees in the COVID-19 pandemic are described in detail [62].

As of May 3, 2020, an interdisciplinary group of experts indicated the possible consequences of restrictive infection prevention measures in nursing homes for the elderly, such as impairment of well-being, increased susceptibility to illness and increased mortality [63]. With reference to human dignity – especially at the end of life – they demanded the retention of familiar surroundings and the possibility of personal accompaniment of the dying person as well as a good farewell by caregivers [63]. Innovative concepts were demanded, in order to achieve a balance between infection prevention and social participation as well as quality of life [64]. 

## Notes

### Competing interests

The authors declare that they have no competing interests.

## Figures and Tables

**Table 1:**
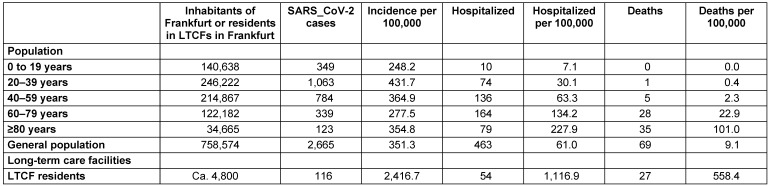
Age-related incidences in the general population and LTCF residents (as of 28.08.2020)

**Table 2:**
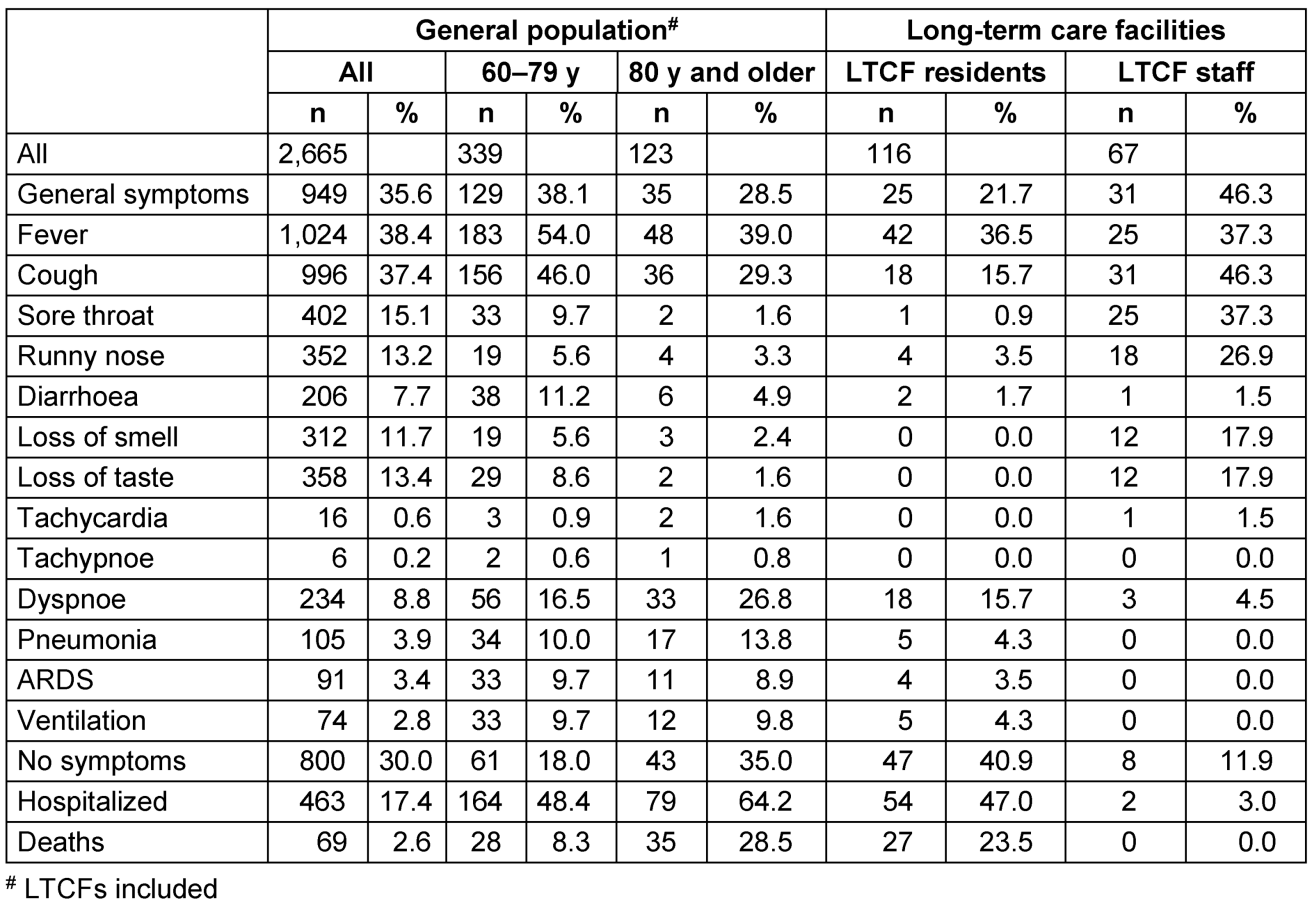
Symptoms in SARS-CoV-2-positive general population and in long-term care residents in Frankfurt am Main (as of 28.08.2020)

**Table 3:**
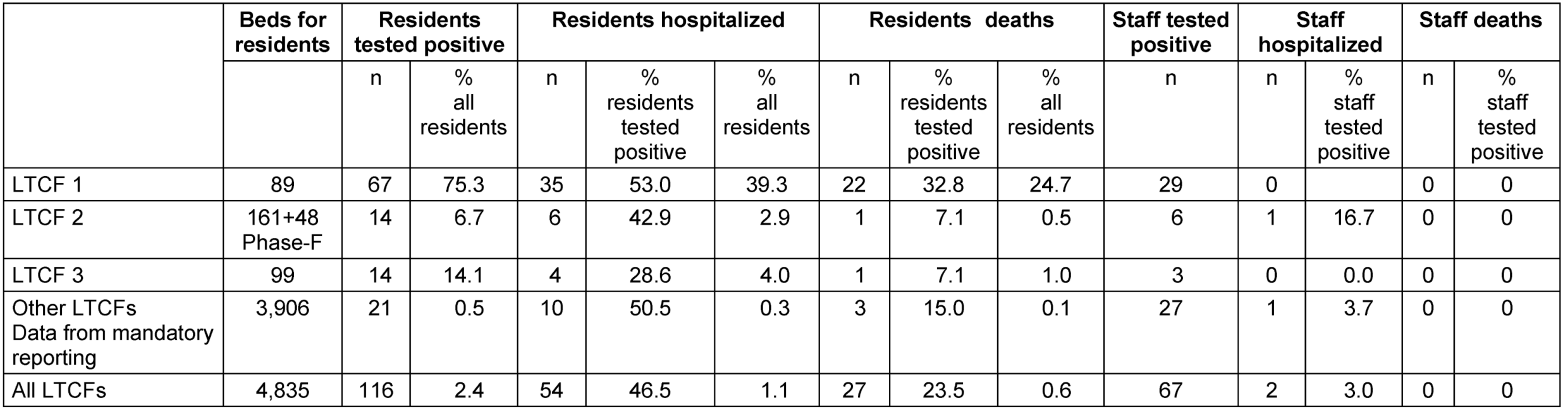
Comparison of COVID-19 infections, hospitalizations, and deaths in resisdents and staff of LTCFs in Frankfurt am Main (as of August 28, 2020)
